# Understanding the Immunology of Normothermic Machine Perfusion

**DOI:** 10.3389/ti.2023.11670

**Published:** 2023-07-19

**Authors:** Menna Ruth Clatworthy, Christopher John Edward Watson

**Affiliations:** ^1^ Department of Medicine, University of Cambridge, Cambridge, United Kingdom; ^2^ Department of Surgery, University of Cambridge, Cambridge, United Kingdom

**Keywords:** liver transplantation, normothermic machine perfusion, single cell RNA sequencing, immunology, liver preservation

Our early experience with normothermic liver perfusion (NMP) led us to identify four compartments that might influence the viability of livers [1]. While we and others have tried to characterize the hepatocyte and cholangiocyte compartments [2–5], and have provided some recent insights into adverse factors in the vascular compartment [6, 7], the immune compartment has remained relatively unexplored, until now. The recent paper by Hautz et al. from Schneeberger’s group in Innsbruck has thrown important light on immune cell changes during the course of perfusion [8]. This work is significant, as a better understanding of the immunological changes occurring during NMP will inform therapeutic interventions to counter reperfusion injury and improve organ quality. Thus, this paper is an important and welcome landmark dataset.

The paper is divided into two parts, analysis of liver biopsies and analysis of cellular components and cytokines in serial perfusate samples. Single cell RNA seq (scRNAseq) was undertaken on eight livers, generating data on all cellular components of liver biopsies, including immune cells within the vasculature and tissue, comparing liver cellular transcriptomes before and at the end of NMP. They showed that nearly half the immune cells in the liver biopsies were neutrophils (defined by *FCGR3B*-expression), a novel observation, likely related to differences in the techniques used to profile cells. The next most frequent immune cell types were monocyte/macrophages (CD68, 8%). They went on to show that the neutrophils emigrate from the liver during NMP, and many appear to be lost from the circuit and do not recirculate to the liver, although they remain the predominant cell type. This is in contrast to macrophages (CD68^+^) and T (CD3 and CD4) and B cells (CD79A) whose proportions did not change significantly. They next considered the transcriptional changes occurring in neutrophils and monocyte/macrophages subclusters during NMP; Neutrophil chemokine receptor expression changed such that cells downregulated *CXCR1/2* and upregulated *CXCR4*, the receptor mediating the return of aged neutrophils to the bone marrow for clearance. Their chemokine profile also changed, with marked expression of *CXCL8*/IL8—the major neutrophil recruiting chemokine—during perfusion, suggesting a capacity for autocrine signalling. The authors propose that overall, the transcriptional changes observed in neutrophils during NMP are consistent with a progression to an aged, chronically activated/exhausted neutrophil phenotype. This suggests that the inflammatory response to reperfusion may be exhausted with time on NMP, an important observation. Neutrophil recruiting chemokines (*CXCL2*, *CXCL8*) were among the most upregulated transcripts in kidneys undergoing NMP [9], suggesting similar processes may also be at play in kidneys.

Four clusters of monocyte/macrophages were identified. The M0 cluster that dominated pre-NMP samples expressed high levels of inflammatory markers such as *S100A8/9*, whilst the M3 cluster enriched in post-NMP samples had a more mixed transcriptome, expressing both pro-inflammatory molecules such as *CTSL* (encoding Cathepsin-L, a protease capable of tissue damage) and anti-inflammatory and tolerogenic molecules (for example, *HMOX1*, encoding haemoxygenase-1, which degrades haem and reinforces an M2 macrophage phenotype), with the potential to counter reperfusion injury.

When Hautz et al. looked at the cellular composition of perfusate in 26 livers, they saw a rapid increase in leucocyte numbers after the start of NMP, predominantly neutrophils, NK cells, B cells and monocyte/macrophages [8]. Numbers peaked at 6 h and thereafter declined. They proceeded to immunophenotype the cells and showed that amongst T cells, the initial predominance of CD4 (48%) and CD8 (44%) at 1 h changed over time, becoming predominantly CD4 cells, with more demonstrating a CD3^+^CD4^+^FoxP3^+^ Treg phenotype.

Finally, they examined cytokine protein levels in the perfusate. As demonstrated by others, many cytokines are secreted into the perfusate over the duration of perfusion. Among cytokines, they identified IL6 as one of interest, as it was increased in DCD grafts compared with DBD grafts, and was higher in discarded livers relative to those transplanted, with macrophages implicated as the major source in the single cell transcriptomic data. We have independently observed similar associations of perfusate IL6 with adverse clinical outcomes, with the highest levels found in a liver suffering primary non function (Watson, unpublished). These data raise the potential of perfusate IL6 as a viability biomarker, but require larger datasets to confirm. The authors also found TNF to be significantly raised in the perfusate of discarded livers compared to transplanted livers, together suggesting that a heightened innate inflammatory response is associated with worse outcomes, and mirroring bulk transcriptomic data in kidneys undergoing NMP [9].

What can we take away from the paper for clinical use? First, potential targets for intervention have been identified. Immune cells are activated at the start of perfusion, cytokines released, and neutrophils, the most dominant cells, exhausted over time. Targeting DAMPs (damage-associated molecular patterns) or cytokines such as IL1β and IL18 capable of stimulating neutrophil activation may mitigate the initial immune response. Elevated IL6 and TNF were associated with worse outcomes, but these associations remain correlative rather than definitively causative. The authors recommend selective neutralization of cytokines, rather than global cytokine removal, which we have shown to reduce the transcriptional changes associated with delayed graft function in kidneys [9].

Even without removal or neutralization of specific cytokines, the data from Hautz et al. suggest that prolonging perfusion over 6 h may in itself result in an environment more conducive to liver recovery, and therefore decisions regarding viability should be delayed, as suggested by other work from the Innsbruck group which showed that lactate values at 6 h are the most predictive value (paper in preparation). In addition, the emigration of neutrophils during NMP, and their loss on the circuit, may result in a less immunogenic graft.

The literature on cardiopulmonary bypass (CPB) reports a systemic inflammatory response syndrome which is associated with high levels of IL8 [10, 11], and is thought to be related to contact activation of neutrophils exposed to the tubing and oxygenator surfaces of the CPB circuit and the sheer stresses involved [12, 13]. These surfaces may account for the sequestering of neutrophils in NMP. Hence it is possible that it is the mechanical circuit, rather than the liver, that is responsible for the transcriptional changes observed in neutrophils in this study. Therefore another therapeutic avenue to explore would be interventions to the circuit, such as “pacifying” the circuit with high density lipoprotein or albumin before perfusion begins to reduce neutrophil adhesion, or using human albumin in place of Gelofusine [14]; alternatively consideration may be given to altering the oxygenator design [15], to assess whether any of these interventions results in less neutrophil activation and sequestration, and cytokine production.

Overall, this study by Schneeberger’s group provides a granular view of cell-specific transcriptional changes occurring in immune populations during liver perfusion, coupled with an assessment of the perfusate, delivering a useful and timely resource for the community and highlights potential therapeutic avenues.
